# Suction-Assisted Laryngoscopy and Airway Decontamination (SALAD) for Emergency Airway Management: A Systematic Review of Evidence and Implementation

**DOI:** 10.3390/jcm14207430

**Published:** 2025-10-21

**Authors:** Saniyah Shaikh, Hamad Hejazi, Safwaan Shaikh, Adeeba Sajid, Rida Shahab, Ayesha Deed, Rida Afnan, Anam Hashmi, Raiyan Ehtesham Ahmed Sharieff, Asfiya Naureen, Marcelo A. F. Ribeiro

**Affiliations:** 1 College of Medicine, Alfaisal University, Riyadh 11533, Saudi Arabia; sanshaikh@alfaisal.edu (S.S.); smshaikh@alfaisal.edu (S.S.); asajid@alfaisal.edu (A.S.); rshahab@alfaisal.edu (R.S.); adeed@alfaisal.edu (A.D.); rafnan@alfaisal.edu (R.A.); ahashmi@alfaisal.edu (A.H.); raiyaneas6161@gmail.com (R.E.A.S.); anaureen@alfaisal.edu (A.N.); 2 Newcastle Upon Tyne Hospitals Trust, Newcastle NE1 4LP, UK; hamad.hejazi@nhs.net; 3 R Adams Cowley Shock Trauma Center, University of Maryland, Baltimore, MD 21201, USA

**Keywords:** airway management, airway visualization, emergency medicine, critical care, suction assisted laryngoscopy and airway decontamination (SALAD)

## Abstract

**Background**: Emergency airway management is a crucial and complex procedure frequently performed in the emergency department (ED). Airway contamination usually caused by blood, secretions, and emesis impairs ventilation, reduces successful intubation, and increases the complication rates, leading to difficult laryngoscopy, delayed intubation, and increased mortality rates. One technique employed to decontaminate these airways when standard approaches fail is Suction-Assisted Laryngoscopy and Airway Decontamination (SALAD). **Methods**: A comprehensive literature search was conducted across PubMed, Cochrane, and Science direct databases following a specific search strategy. All search results were screened in a two-stage process (title–abstract and full-text screening) in accordance with Preferred Reporting Items for Systematic Reviews and Meta-analyses (PRISMA) 2020 guidelines. Data from finalized articles were extracted using a standardized excel file developed a priori. Lastly, quality and risk of bias were assessed using appropriate tools according to respective study designs, and data were narratively synthesized. **Results**: A total of 224 records were identified. Upon screening, seven studies were included consisting of five simulation-based studies and two clinical case reports. Simulation studies reported that SALAD training significantly improved first-pass intubation success (53.7–90.2%), reduced time to intubation (up to 30 s), and enhanced airway visualization. Clinical cases further reported successful first-pass intubation in patients with massive airway contamination without complications. Overall, across both study types, the SALAD technique improved airway management performance, provider confidence, and airway contamination control compared to standard suction techniques. **Conclusions**: This systematic review highlights the benefits of the SALAD technique by enhancing airway visualization, reinforcing it as a significant tool for contaminated airway management. Trainees who underwent SALAD training demonstrated improved first-pass intubation success, reduced intubation time, and increased operator confidence. While data from the included studies seems promising, most studies are small simulation-based studies with limited clinical outcome data. Given its clinical relevance and educational value, future studies must prioritize high-quality research in clinical environments to establish SALAD’s efficacy and to define its role in integration into prehospital protocols.

## 1. Introduction

Emergency airway management is a crucial and complex procedure frequently performed in the emergency department (ED). In spite of its significance in research and clinical settings, emergency airway management in the ED is often challenging due to contamination of the airway by vomitus, blood, secretions, facial trauma, and chest compression during resuscitation. One of the primary aims in emergency airway management is first-pass success, which is directly linked with improved patient outcomes and is crucial for preventing adverse effects [1]. A prospective study evaluated 53 patients undergoing prehospital intubation, out of which 18 patients’ (34%) airways were contaminated with vomitus and blood [2]. Vomiting and regurgitation are frequently encountered in outpatient cardiac arrest patients with an incidence of 20–30%, which highlights the frequent occurrence of airway contamination, impaired visualization, and ultimately difficulty with smooth intubation [3]. Moreover, contamination of the airway plays a critical role in simulation training, more specifically, in cadaveric airway management training (CAMT), which is an essential skill to regarding not causing the patient risk, and techniques like Suction-Assisted Laryngoscopy and Airway Decontamination (SALAD) have been developed to address these challenges [4,5].

Contamination of the airway from blood, secretions, and emesis impairs the ventilation process, reduces success, and increases complication rates, leading to difficult laryngoscopy, delayed intubation, and increased mortality rates. According to the National Audit Project 4 (NAP4) Report, one of the most common causes of mortality was aspiration of gastric contents. Moreover, Can’t Intubate, Can’t Oxygenate (CICO) cases were also reported regardless of the use of direct or video laryngoscopy, undermining the need for techniques like SALAD [6]. The traditional approach to the contaminated airway involves aspirating the airway and repositioning the patient. Indeed, many of these methods, like video laryngoscopy, can be challenging at times, such as when the lens could be obstructed by the presence of blood or vomitus, impairing visualization [7]. Furthermore, in a randomized manikin study (SATIATED), they investigated how standard approaches had a 53.7% success rate, whereas after a brief training of the SALAD technique, success rose to 90.2%, which demonstrates the need for airway decontamination techniques like SALAD [2,3]. Shortages in essential equipment, medications, and trained individuals might hinder effective airway management, leading to suboptimal care and high rates of complications, exacerbating challenges [8].

SALAD, developed by Dr. James C. DuCanto, helps to manage massively contaminated airways when the standard approaches fail. It involves a rigid suction catheter (RSC), which is gripped overhand to clear the fluids ahead of the laryngoscopy, thereby protecting visualization and first-pass success. For the continuous clearing of the hypopharynx, even if there is active airway contamination, the “SALAD Park” approach is utilized where the RSC is removed and re-inserted to the other side of the laryngoscope to ensure smooth visualization of the esophageal opening [6]. Additionally, SALAD is considered more effective across various airway devices and simulation models, including low-cost and highly reliable trainees [9,10,11].

**Figure 1:**
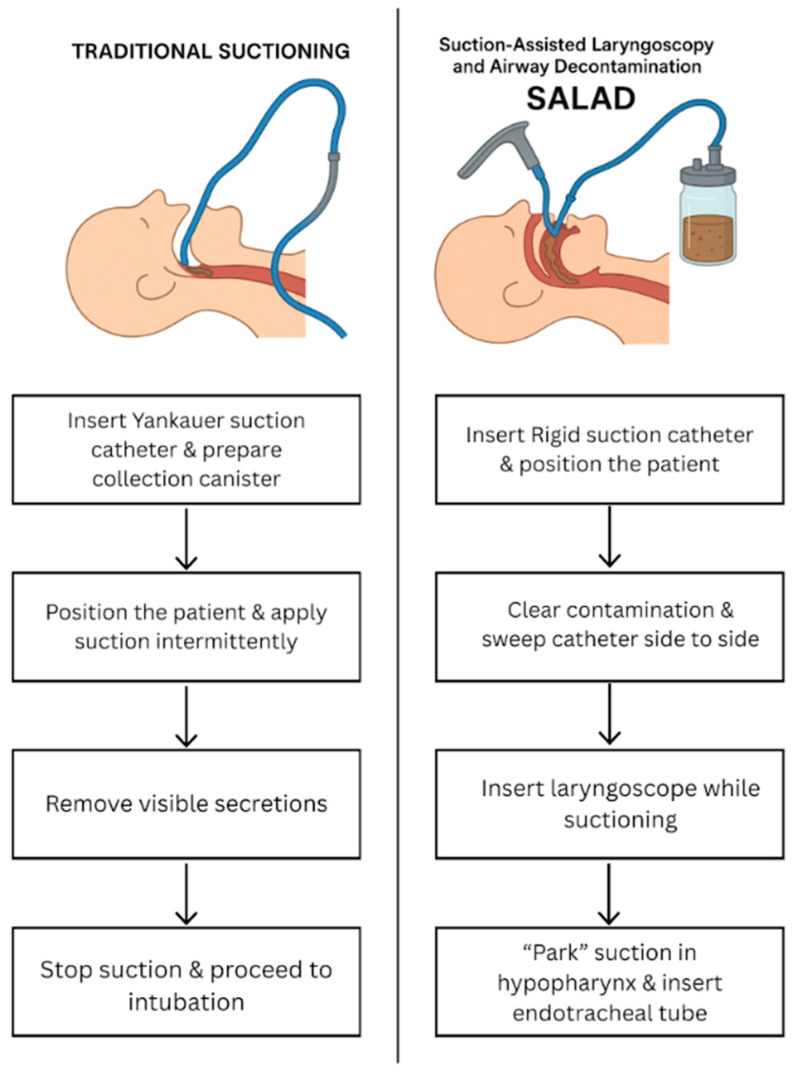
Comparison of traditional suction technique vs. SALAD technique, [9,10].

Unlike standard approaches, which rely on intermittent suctioning and a lack of specific training, SALAD has simultaneous suctioning and laryngoscopy and offers better visualization and decontamination, making it beneficial for high-risk cases in the ED. Given the increasing importance of airway contamination during emergency intubation, this systematic review aims to assess the effectiveness of the SALAD technique in improving intubation success rates, time to intubation, and quality of airway visualization. Additionally, it will also evaluate the safety and potential complications associated with SALAD use in clinical and simulation settings. Furthermore, this review will identify knowledge gaps in current evidence and highlight areas for future research for SALAD use in emergency and anesthesiology airway management. Lastly, it will compare the reported effectiveness of the SALAD technique with other airway decontamination strategies used in emergency and anesthesiology settings. with an overview presented in Figure 1.

## 2. Methods

### 2.1. Protocol Registration

Methodological details ensuring the integrity of this systematic review were predefined and registered to PROSPERO (CRD420251075145).

### 2.2. Eligibility Criteria

Inclusion Criteria:

Studies were eligible if they involved patients of any age (neonates to the elderly) undergoing airway management in emergency, anesthesia/perioperative, critical care, or simulation settings. The intervention had to include the utilization or evaluation of the SALAD technique. Comparators, when present, included conventional suction, airway management methods, or alternative decontamination techniques. Eligible study designs were randomized controlled trials (clinical or simulation), non-randomized interventional studies, observational cohorts (prospective or retrospective), case series, and case reports. Only English-language publications were included.

Exclusion criteria:

Studies were excluded if they involved healthy volunteers without SALAD application, lacked SALAD intervention or outcome data, or were animal/laboratory studies without clinical or simulation relevance. Reviews, meta-analyses, expert opinions, commentaries, conference abstracts, book chapters, protocols, guidelines, and position statements were also excluded. Non-English language studies were not considered.

### 2.3. Search Strategy

A comprehensive literature search was conducted on 19 June 2025 in PubMed, Cochrane, and Science direct databases without search date restrictions. The following search strategy was applied:

Pubmed and Cochrane:

(“Suction Assisted Laryngoscopy and Airway Decontamination” OR SALAD OR “airway decontamination”) AND (“airway management” OR intubation OR laryngoscopy OR “emergency airway” OR “emergency intubation”) AND (emergency OR anesthesiology OR anesthesia OR perioperative OR “critical care”).

Science Direct (modified due to platform limitations):

(“SALAD” OR “Suction Assisted Laryngoscopy and Airway Decontamination”) AND (“airway management” OR intubation OR “laryngoscopy”) AND (emergency OR anesthesiology OR perioperative).

### 2.4. Study Selection

All search results were screened in a two-stage process in accordance with Preferred.

Reporting Items for Systematic Reviews and Meta-analyses (PRISMA) 2020 guidelines. Title and abstract screening was performed independently by two reviewers (S.S. and A.D.), followed by full-text screening independently conducted by two different reviewers (R.S. and R.S.). Any disagreements were resolved through discussions between reviewers until a consensus was reached.

### 2.5. Data Extraction

Data extraction was performed using a standardized Excel file, developed a priori based on the PICOS framework by two authors (A.N. and S.S.). The form captured information on study identifiers (ID, DOI, authorship, publication year, title), study characteristics (country/institution, funding source, declared conflicts of interest, study design, setting, simulation, or clinical nature, and details regarding blinding or randomization). Further data was extracted on population characteristics, including participant type, total number of participants, adherence or completion rates, and target patient type. Intervention data focused on the use of the SALAD technique, including whether SALAD was implemented, its implementation type, devices/equipment used, whether SALAD training was included, training content, duration, and the type of instructor involved. Comparator details were recorded, including the design and description of comparator interventions and the provider experience associated with the comparator. Outcome measures (primary and secondary) included time to successful first-pass intubation and timing of measurement, quality of visualization, associated timing, aspirate volume or airway contamination and timing, reported adverse events and their timing, success rate in soiled airways, number of intubation attempts, skill retention at follow-up, and pre/post-confidence improvement measures. Quantitative results, statistical significance, and any subgroup analyses were documented. Additionally, qualitative feedback was extracted, including provider feedback, confidence scores, perceived usefulness and realism of the simulation or intervention, and reported challenges. The risk of bias and study quality were assessed using standardized appraisal tools, with extracted data including tool type, risk of bias score or summary, and noted limitations. Reviewer comments, relevance to the review objective, and any other techniques compared were also documented. Three authors (R.S., R.A. and A.H) performed data extraction with discrepancies resolved through discussion with the senior author (S.S.).

### 2.6. Quality Assessment

The methodological quality and risk of bias of included articles were assessed using the appropriate tools based on the study design. Randomized controlled trials (RCTs) were appraised using the ROB2 tool, non-randomized studies with the Robins 1 tool, and case reports using the CARE checklist. For the ROB2 tool, the risk of bias judgment was categorized as “low risk”, “some concerns”, or “high risk”, and for the Robins 1 tool, the judgment was categorized as “moderate risk”, “serious risk”, or “critical risk” to promote transparency and consistency in evaluating the internal validity of the studies. The CARE checklist assigns one point per fulfilled criterion (maximum 13), with scores >11 indicating low risk, 7–11 indicating moderate risk, and ≤6 indicating high risk of bias. Quality assessments were conducted independently by three reviewers (A.S., A.H. and H.H.) who divided the studies among themselves. Any discrepancy in scoring or interpretation between primary reviewers was resolved by discussion with the third reviewer to ensure methodological rigor across studies.

### 2.7. Data Synthesis

Data was synthesized narratively and descriptively to address the study objectives, highlighting themes, differences, and methodological limitations across studies.

### 2.8. Certainty of Evidence Assessment

The certainty of evidence for study outcomes was assessed using the GRADE (Grading of Recommendations Assessment) approach. The assessment evaluated five domains: ROB, inconsistency, indirectness, imprecision, and publication bias. Outcomes were downgraded by one level if serious concerns were identified in a domain or by two levels for every serious concern. Narrative and quantitative synthesis for each outcome informed judgments for inconsistency, indirectness, imprecision, and potential publication bias.

## 3. Results

### 3.1. Study Selection Description

**Figure 2:**
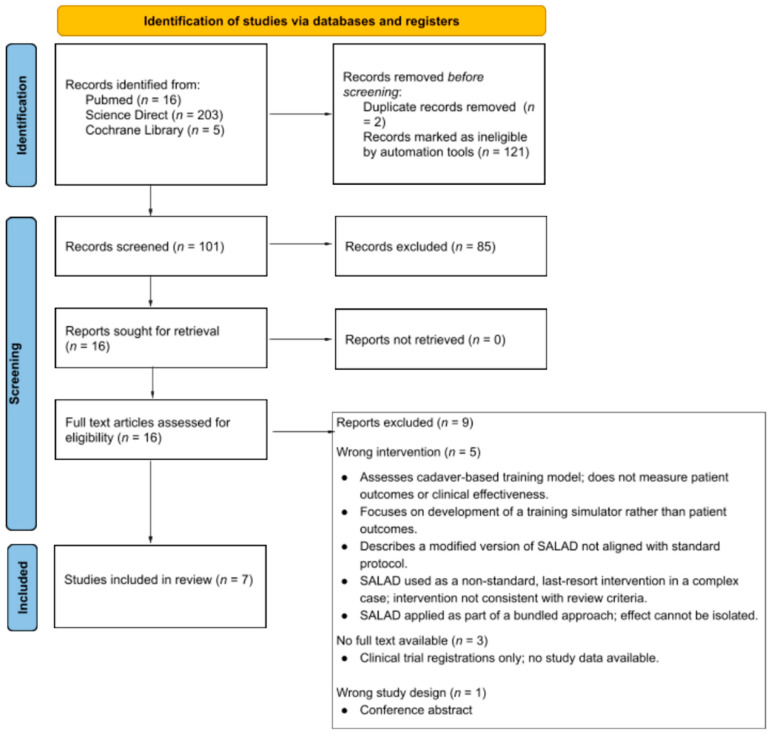
PRISMA 2020 flow chart depicting the study selection process.

A prespecified search from PubMed, Cochrane Library, and ScienceDirect databases identified 224 studies. Two duplicate records were identified and removed. The use of automation tools further marked the exclusion of 121 articles as being ineligible, reducing the number of studies for screening to 101. After title and abstract screening, 85 studies were excluded, leaving 16 studies for full-text screening. Of these, nine studies were excluded due to three studies being clinical trial registrations lacking full text, five with the wrong interventions, and one with the wrong study design. Ultimately, seven articles were included in the final review. The overall study selection process is illustrated in the PRISMA flow diagram (see Figure 2).

### 3.2. Study Characteristics

A total of seven studies were included in this review, comprising five simulation-based investigations and two clinical case reports. The included studies were conducted in diverse settings, ranging from helicopter emergency medical services and paramedic training programs to emergency departments in the United States, the United Kingdom, and Taiwan. Participants included a wide spectrum of providers such as paramedics, emergency medicine technician–paramedics, resident doctors, anesthesiologists, emergency physicians, nurses, and respiratory therapists. The study designs encompassed randomized controlled manikin trials [3,12], prospective before–after educational interventions [13,14], observational simulation studies [15], and clinical case reports [16,17].

Across the simulation studies, SALAD training was consistently associated with improved intubation outcomes when compared with either baseline performance or conventional suctioning methods. For instance, a USA prospective before–after study among 20 HEMS crew members demonstrated a progressive reduction in intubation times, with median values decreasing from 60.5 s pre-training to 43.0 s immediately post-training, and further to 29.5 s at three-month follow-up, thereby confirming skill retention [13]. Similarly, a randomized crossover manikin trial in the UK showed that first-pass success increased markedly from 53.7% to 90.2% with SALAD training, while mean intubation time decreased by 11.7 s (*p* < 0.001) [3]. Moreover, a pilot before–after study involving 41 EMT-paramedics in Taiwan reported a significant improvement in intubation success rates (from 82.9% to 92.7%), alongside a reduction in median intubation time (37.1 s to 26.9 s, *p* = 0.031), and a striking increase in provider confidence from 22.0% to 97.6% [14]. In addition, an experimental manikin study in the USA compared three airway decontamination strategies: traditional rigid suction, intentional esophageal intubation, and SALAD, finding that SALAD achieved the highest first-pass success (97% vs. 91% and 88%, respectively), with significantly better laryngeal visualization and shorter intubation times (*p* < 0.05) [12]. Furthermore, an observational simulation study introducing the SALAD simulator to USA emergency and critical care providers found substantial gains in perceived realism and confidence, though objective performance metrics were not reported [13].

The clinical evidence, though limited, provided complementary real-world support. A prehospital case report from the USA involving an adolescent trauma patient with active intraoral hemorrhage demonstrated that SALAD, combined with video laryngoscopy, enabled successful first-pass intubation without aspiration, hypoxia, or hemodynamic compromise [16]. Likewise, in an emergency department case of a woman with massive regurgitation and respiratory distress, the use of SALAD allowed for rapid suctioning, clear glottic visualization, and uncomplicated intubation without desaturation or aspiration [17].

Overall, the included studies consistently highlighted that SALAD was feasible, effective, and superior to conventional suctioning approaches in both simulated and clinical massively contaminated airway scenarios. A detailed summary of the study characteristics is presented in Table S1.

### 3.3. Reporting Risk of Bias

A robust risk of bias assessment was carried out for all included studies using design-appropriate tools. Randomized controlled trials were assessed for risk of bias via the RoB-2 tool, observational or comparative studies were assessed via ROBINS-I, and case reports were assessed via the CARE checklist. This process was carried out independently by two peers, enhancing the effectiveness of the risk of bias assessment. Conflicts were resolved appropriately and led to an evidence-based insight into the bias of these studies. Simulation teaching-based observational studies, showing promising results as per the previous section, landed a higher risk of bias as per the ROBINS-I tool used for their assessment. A US-based observational study using simulation had the best risk of bias outcome, which was still a serious risk of bias [15]. Before–after simulation studies came up with a critical risk of bias as per ROBINS-I [13,14]. Confounding was found to be a key cause of the increased risk of bias in the observational studies.

The randomized crossover manikin studies show SALAD in a promising light, and their risk of bias assessment was supportive as well. As per ROB-2, they each carried a lower risk of bias. This can be due to the fact that randomized crossover manikin studies reflect a better study design to support the evidence for SALAD. Case reports were at a higher risk of risk of bias, as assessed by the CARE case study quality checklist. One case study showed a higher quality of case reporting, which translated well into its risk of bias assessment [16].

**Figure 3:**
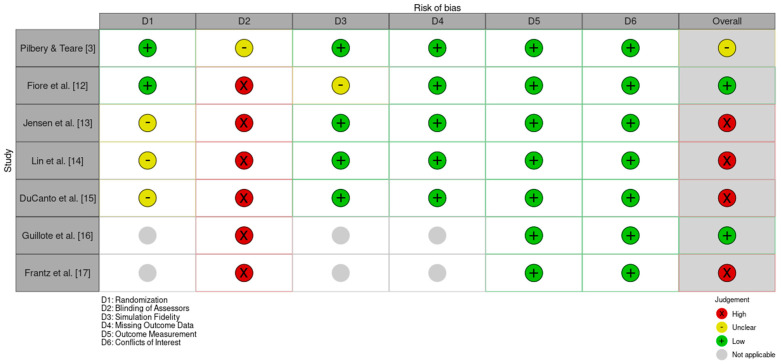
Summary of risk of bias across included studies [3,12,13,14,15,16,17].

With a widespread range of conclusions about individual studies’ risk of bias within this body of evidence, it carries a moderate-to-high risk of bias primarily due to confounding within observational studies and a ~28% reliance on case studies of varying quality. The results should be interpreted cautiously so as to account for the observational and descriptive nature of a majority of the evidence. The Pilbery & Teare [3] study demonstrates the best piece of evidence supporting the SALAD technique, alongside having a low risk of bias. See Figure 3. for summary of risk of bias across included studies.

### 3.4. Outcomes

#### 3.4.1. Primary Outcomes

Primary outcomes analyzed included first-pass intubation success, time to intubation, quality of airway visualization, aspirate volume, timing of aspirate volume measurement, adverse events reported, and success rate in soiled airways.

##### Success Rate in Soiled Airways

**Figure 4:**
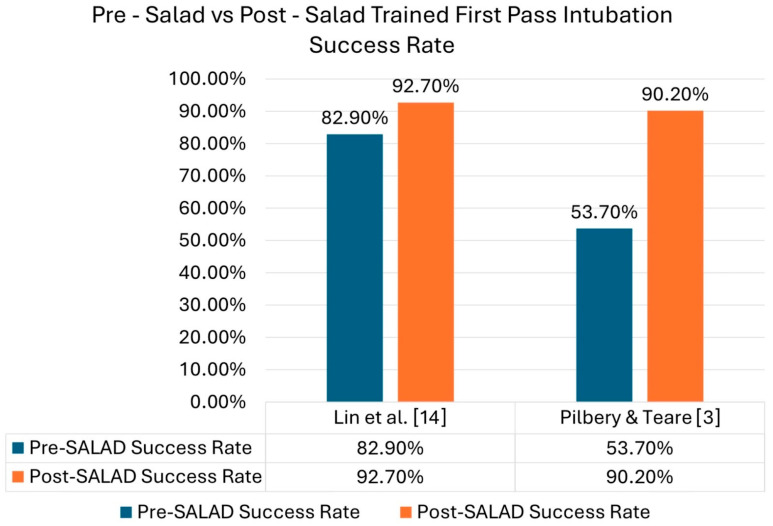
Pre-SALAD vs. post-SALAD trained first-pass intubation success rate [3,14].

Intubation success improved significantly across all simulation-based studies following SALAD training. In the SATIATED trial [3], first-pass success rose from 53.7% to 90.2% (*p* < 0.001) after SALAD instruction. Fiore et al. reported an overall success rate of 97%, with SALAD showing faster intubation times and higher success rates (*p* < 0.05) [12]. There was improved success from 82.9% to 92.7% after training (*p* < 0.05) [14]. Increased provider confidence post-training was reported by Ducanto et al. [15]. This trend is illustrated in Figure 4, which shows consistent improvement across studies before and after training. Furthermore, there were improvements in reported median times [13].

For clinical studies, both case reports involving real-world SALAD use achieved first-pass intubation success. An adolescent trauma case patient experiencing intraoral hemorrhage was successfully intubated without any complications [16]. Similarly, in the emergency department case, SALAD enabled immediate visualization and successful intubation in a regurgitating patient [17].

##### Time to Intubation

**Figure 5:**
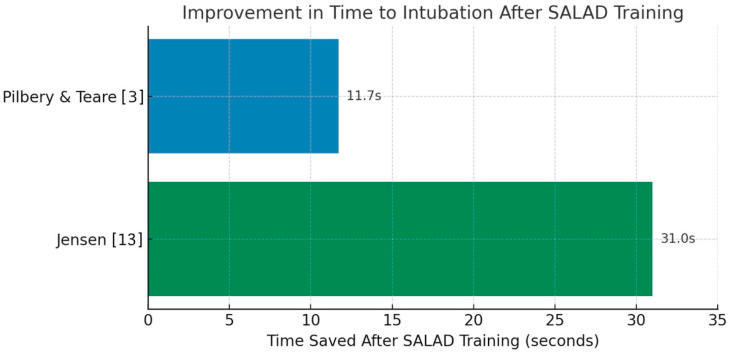
Reported improvement in time to intubation before and after Salad training [3,13].

Time to intubation significantly decreased following SALAD training. As demonstrated in Figure 5, reductions were consistently observed with training, leading to faster intubation times. A reduction in median intubation time from 60.5 s pre-training to 43.0 s post-training, and 29.5 s at 3-month follow-up (*p* < 0.01) was observed [13]. Supporting this, Lin et al. reported median intubation time improvement from 37.1 s to 26.9 s (*p* = 0.031) [14]. Similarly, the SATIATED trial reported a mean time saving of 11.7 s in SALAD-trained participants compared to controls (*p* < 0.001) [3]. In the manikin comparison by Fiore et al., SALAD performed favorably compared to standard suction and intentional esophageal intubation (IEI), although raw time values were not consistently reported [12].

Neither of the case reports included precise time-to-intubation metrics. However, both authors noted that SALAD enabled rapid airway clearance and prompt intubation in challenging settings [16,17].

##### Visualization Quality

SALAD training enhanced glottic visualization across studies. While formal grading systems such as Cormack–Lehane were not universally applied, multiple studies reported improved visualization. DuCanto et al. noted increased realism and visual clarity using the SALAD simulator, despite not measuring specific grades [15]. The trials conducted by Pilbery et al. showed participants reporting improved visual fields and reduced airway contamination, contributing to the improved intubation success [3]. Subjective rating was used in Fiore et al., and participants showed better visualization quality with SALAD [12]. Lin et al. documented qualitative improvements in visibility, confirmed through participant feedback [14].

In both real-world cases, SALAD facilitated rapid glottic visualization despite active contamination. Frantz et al. emphasized that visualization improved immediately after the SALAD sweep, allowing smooth tube passage [17]. Guillote et al. similarly described enhanced visibility that was essential in the bloody airway scenario [16].

##### Aspirate Volume

While most studies did not provide exact measurements of aspirated fluid or the timing of suction, there was consistent qualitative evidence supporting the SALAD technique’s ability to manage airway contamination. Pilbery et al. noted that after training, there was visibly less regurgitation obstructing the airway during intubation [3]. There was improved control over contaminated airways, with better use of suction and handling of secretions [14]. Fiore et al. compared three suction strategies and found that SALAD offered the most effective airway clearance, though specific aspirate volumes were not specified clearly [12].

The clinical studies did not include data on aspirate volume but highlighted the rapid clearance of airway decontamination [16,17].

##### Safety and Complications

Adverse events could not be measured directly in manikin studies. However, Lin et al. reported no simulated complications post-training and noted improved confidence in contamination control [14]. The SATIATED trial emphasized that SALAD reduced regurgitation pooling and improved contamination control without introducing procedural difficulty [3].

Neither of the two case reports reported aspiration, hypoxia, airway trauma, or hemodynamic instability. Both authors described the SALAD technique as effective in mitigating risk in soiled airway scenarios. Guillote et al. highlighted that SALAD allowed controlled, complication-free intubation in a high-risk trauma context [16]. Frantz et al. similarly noted the absence of complications during and after intubation [17]. See Table 1, which presents a comparative summary of the key primary outcomes.

#### 3.4.2. Secondary Outcomes

Secondary outcomes measured included number of attempts, skill retention at follow-up, and provider confidence improvement pre- and post-training.

##### Number of Attempts

The number of intubation attempts was not consistently reported across all studies. However, some simulation studies suggested a reduction in attempts following SALAD training. Lin et al. noted that participants who were initially unable to intubate before training were able to successfully intubate after training [14]. The SATIATED study showed a higher rate of first-pass success after the intervention reduced the number of attempts for intubation [3]. Fiore et al. also reported improved overall success rates with the SALAD technique compared to other suction methods, suggesting that fewer attempts were needed [12].

Both clinical case reports involved successful intubation on the first attempt when using SALAD, supporting the simulation findings that the technique may reduce the need for multiple attempts [16,17].

##### Skill Retention

Skill retention was specifically addressed in one study. Jensen et al. assessed participants three months after the initial SALAD training and observed improvements in success rates and intubation times, which were maintained at follow-up (*p* < 0.001) [13]. This shows that the effects of training with the SALAD technique are not limited to short-term performance. In SATIATED, skill retention was not measured directly, but consistent post-training showed sustained benefits [3]. No follow-up data were available in the remaining simulation or clinical studies, so long-term skill retention in real-world settings remains unclear.

##### Provider Confidence

Provider confidence increased with the use of the SALAD technique. Jensen et al. reported statistically significant improvement in confidence scores post-training, which were sustained at 3-month follow-up (*p* < 0.001) [13]. DuCanto et al. described positive learner feedback and greater engagement, though formal scores were not recorded [15]. Lin et al. noted that 75.6% of trainees gave the highest satisfaction rating regarding the training [14]. SATIATED included qualitative participant feedback, reflecting improved control and confidence [3].

Confidence was not directly assessed in the clinical studies, but both case reports described confident execution of SALAD in high-stress airway scenarios [16,17]. See Table 2, which presents a comparative summary of the key secondary outcomes.

### 3.5. Certainty Assessment

The certainty of evidence evaluated for primary and secondary outcomes was very low. This was primarily due to a serious risk of bias in all of the studies (observational simulation and case reports), indirectness from simulation-based evidence compared with clinical populations, and imprecision due to small sample sizes. Despite these limitations, consistent improvements were observed in first-pass intubation success, time to intubation, visualization quality, and provider confidence, suggesting that SALAD may be effective. Nevertheless, the very low certainty underscores the need for higher-quality clinical studies to validate these findings. See Table 3 for a summary of the findings and the certainty of the evidence.

## 4. Discussion

This systematic review looked into the strength of evidence, effectiveness, and the advantages of the SALAD technique in managing contaminated airways. Across simulation and clinical-based studies, SALAD consistently demonstrated value in shorter intubation times, improved first-pass success, better airway visualization, and increased provider confidence.

The SALAD technique has been studied alongside other airway management strategies for use in contaminated airway scenarios. In both real-world and simulation applications, SALAD has proven to be either equivalent or more effective than traditional suction methods and other techniques such as intentional esophageal intubation (IEI). Fiore et al. ran a randomized study comparing traditional suctioning, intentional esophageal intubation, and SALAD using manikins. While all three techniques were effective in securing the airway, SALAD and IEI were more effective in minimizing airway contamination. Participants performing the SALAD technique reported increased confidence and capability following the simulation, suggesting that the technique is a valuable tool for training providers to handle high-risk airway emergencies [12]. SALAD training also concluded a successful first-pass intubation rate and decreased time to intubation, as reported by Pirotte et al. in prehospital care providers helping those who struggled with contaminated airway scenarios [13].

SALAD provides key technical benefits by incorporating a large-bore suction catheter as compared to other suction methods. Studies evaluating catheter types have shown that traditional Yankauer suction devices tend to clog and struggle with high-volume emesis scenarios due to lower flow rates. In contrast, large-bore catheters such as the DuCanto catheter, standard in the SALAD protocol, are built for contaminated airways and offer stronger suction. The DuCanto catheter consistently outperforms Yankauer devices in cases involving thick or high-volume fluids. This is because of its design, which keeps fluid mechanics in mind, offering improved flow and resistance to clogging [18,19]. In the study by Ko et al., SALAD in combination with DuCanto suction and video laryngoscopy was tested during a simulated massive emesis with improved intubation success (100% vs. 90%, *p* = 0.043) and lower aspirate volume in the bronchi (23.2 mL vs. 40.4 mL, *p* = 0.027), as compared to the standard suction method [6]. Rouleau et al. further illustrated the versatility of SALAD in clinical practice with the use of a DuCanto catheter to direct a bougie during hyper angulated video laryngoscopy, successfully preserving visualization in an extensively soiled airway [20]. The DuCanto catheter offers significant benefits in terms of efficiency and success rates, as per our findings. This raises interesting questions to be explored further regarding comparisons between various medical devices used in the intubation process.

IEI is a technique that involves deliberately placing an endotracheal tube into the esophagus to divert stomach contents and clear the mouth. We took this method into consideration as we explored evidence of alternative techniques. A few case reports suggest IEI may work, but there is yet to be statistically significant evidence regarding its clinical advantages [21,22]. Furthermore, IEI involves intentionally placing the endotracheal tube in the esophagus and then repositioning it, which can add an unwanted increase in the risk of procedural complications. In contrast, SALAD maintains continuous suction during a single intubation attempt, potentially shortening the procedure and reducing airway trauma [12].

Overall, compared to both traditional suction methods and other airway-clearing techniques, studies show that SALAD performs better. Although more clinical trials are needed, existing simulations and case reports suggest that SALAD is one of the most effective techniques for managing contaminated airways [12].

Based on this review, the SALAD technique emerges as a useful and convenient solution in managing airways that are problematic in prehospital critical care scenarios [13]. Its use cases are especially pronounced in cases where the patient is actively vomiting or bleeding, and this undoubtedly paints it as a valuable adjunct to conventional interventions. The SALAD technique seems to work well alongside existing techniques and apparently reduces median time to intubation for participants compared to their pre-training baseline times [16]. This might be partly due to improved visualization and reduced fluid obstruction in airways [13]. The cost, when compared to the relative perceived improvement in patient outcomes, makes the SALAD technique accessible for general adoption. Its relevance also extends beyond clinical scenarios, as the literature shows great benefits of the technique in simulation-based training programs [15]. Adding this technique to standard airway training programs for resident doctors, paramedics, and emergency care providers could build confidence and competence in managing complex airway cases. The evidence provided by our review supports SALAD’s clinical relevance and suggests that it may fill a critical gap in contaminated airway management strategies [13].

Recent research on the SALAD technique offers several notable advantages, both technical and teaching-related strengths, making it a useful tool in airway management training. To begin with, the SALAD technique has been studied using a range of study designs, enhancing the credibility of the evidence. These include randomized trials, such as the SATIATED study, which demonstrated improved first-pass success and faster intubation times among paramedics following SALAD training in a simulated contaminated airway setting [3]. Similarly, observational studies like that of Lin et al. found that structured SALAD instruction showed improvements in the suction technique and intubation effectiveness [14]. Simulation-based training described by DuCanto et al. demonstrates that the SALAD technique closely mimics the difficulty of managing airways contaminated by emesis, thereby helping providers for real-world applications in high-stress airway emergencies. Studies include participants from a wide range of clinical roles, including paramedics, physicians, nurses, and flight medics, highlighting the technique’s flexibility and potential for widespread implementation in both prehospital and hospital environments [15]. Additionally, some research has evaluated whether SALAD’s benefits persist over time. For example, Jensen et al. found that three months after initial training, increases in intubation ability persisted, suggesting that SALAD training may help people retain abilities over time [13]. Finally, SALAD training is supported by high-quality simulation tools. A manikin designed specifically for SALAD training, as reported by DuCanto et al., allows learners to practice intubation in the context of persistent emesis, thereby enhancing their ability to manage difficult, contaminated airways in real-world scenarios [15]. Taken together, the range of studies, broad provider inclusion, evidence of skill retention, and use of realistic simulation models all support the SALAD technique as a reliable and effective training approach for complex airway management.

Keeping in mind the positives and use cases that arise from SALAD, certain limitations have to be considered. Most of the current evidence supporting the SALAD technique comes from simulation-based articles and not from clinical scenarios and case studies [13,14]. Simulation can be useful in recreating complex scenarios in a low-risk environment but does not adjust for real-world uncertainty and complex patient dynamics. This hinders generalization and affects the degree to which these studies can be accepted as standard. Another limitation of the literature reviewed was the relatively small sample sizes of the studies, which increased the risk of bias and made the studies less statistically significant. One limitation that affects the possibility of statistical analyses on the SALAD technique is the heterogeneous nature of the study designs that the review looked at. In addition to this, studies failed to look at the long-term patient outcomes, such as aspiration-related subacute and chronic complications, comorbidities, and critical care stays. Finally, there was no standardization in methods of teaching the SALAD technique in these studies, leading to inconsistencies in the reported effectiveness [13]. These limitations are to be kept in mind, and the current literature is to be approached cautiously while seriously evaluating the SALAD technique for generalized real-life patient scenarios.

The SALAD technique clearly seems promising in terms of efficiency, but as of now, there exist numerous gaps in the current evidence base. Currently, there is a lack of real-world large-scale clinical studies that test its safety, efficacy, and improvement in patient outcomes [17]. This review calls future researchers to prioritize prospective trials in prehospital emergency settings to assess its application and utility during active airway events. This review also identifies that the SALAD technique and its training lack adequate standardization strategies, and this gap should be targeted by future researchers and study designers. These inconsistencies can be resolved by developing and validating a structured curriculum alongside clearly standardized performance metrics [16]. Comparative studies will also be useful to assess SALAD against other novel airway decontamination techniques to statistically analyze relative advantages or disadvantages. Finally, this review calls for researchers to look at long-term patient outcomes such as subacute and chronic patient complications, survival rates, and critical care stays post-intervention. Addressing these research gaps would be crucial towards achieving a better understanding of the SALAD technique’s utility, building a reliable evidence base, and supporting SALAD’s adoption as a frontline intervention.

Looking at the data collected from this review, it is clear that SALAD is a promising approach for managing contaminated airways, particularly in emergency and prehospital settings [15]. Combining suction alongside laryngoscopy addresses a challenging concern in scenarios where airway management is hindered by active nemesis or bleeding, leading to obscured vocal cords and hence making intubation difficult. Although mostly simulation-based, current evidence shows SALAD’s potential to improve visualization and enhance chances of successful intubation, clearly serving as a valuable educational tool to train healthcare providers. Its adaptability across various clinical environments, in addition to its technical utility, makes the SALAD technique stand out [17]. For this technique to be validated and truly become evidence-backed, this review once again calls for further high-quality research in clinical environments. Until then, SALAD should be viewed as an innovative and potentially transformative adjunct to airway management that is worthy of inclusion in training programs and clinical protocols, but with the understanding that its evidence base is still evolving.

## 5. Conclusions

The evidence reviewed shows that the SALAD technique improves the success of intubation in contaminated airways. It increases first-pass success rates, reduces intubation time, and improves airway visualization and operator control when compared to traditional suction methods. While most data come from simulation studies, SALAD consistently outperforms standard methods and provides technical benefits that support its use in emergency and prehospital airway management. Until stronger evidence is available, SALAD may be viewed as a novel approach to airway management, appropriate for inclusion in training and practice, while its clinical evidence remains preliminary.

## Figures and Tables

**Table 1:** Comparative table of key primary outcomes.

Primary Outcome	Pilbery et al. [3]	Fiore et al. [12]	Jensen et al. [13]	Lin et al. [14]	DuCanto et al. [15]	Guillote et al. [16]	Frantz et al. [17]	Conclusion
Time to First-Pass Intubation ↓	✔ *p* < 0.001	✔ *p* < 0.05	✔ *p* < 0.001	✔ *p* = 0.031	✖	✔ (clinical case)	✖	Time to first-pass intubation significantly decreased.
Visualization Quality ↑	✔ *p* < 0.001	✔ (subjective, *p* < 0.05)	✖	✖	✖	✔ (subjective)	✔ (subjective)	Airway visualization quality generally improved, though mostly subjective.
Aspirate Volume/Airway Contamination ↓	✔ *p* < 0.001	✔ (visual, *p* < 0.05)	✖	✔ (visual, *p* < 0.001)	✖	✔ (observed effective)	✔(descriptive)	Consistently reduced airway contamination but objective measurement is rare.
Adverse Events Reported	✖ (None)	✖	✖	✖	✖	✖ (None)	✖ (None)	No adverse events reported.
Success Rate in Soiled Airway ↑	✔ 97.4%, *p* < 0.001	✔ 97%, *p* < 0.05	✔ 100% post-training	✔ 92.7%, *p* = 0.001	✖ (not assessed)	✔ (1st pass, clinical)	✖	Success rate in soiled airways significantly improved.

(✔ = Outcome improved significantly (based on reported data). ✖ = Outcome not improved, not assessed, or not statistically significant. “↑/↓” indicates the direction of change. ↑ = Desired outcome increased (e.g., success rate, confidence). ↓ = Desired outcome decreased (e.g., intubation time, contamination). Where available, *p*-values or descriptive notes are included for clarity on statistical significance).

**Table 2:** Comparative table of key secondary outcomes.

Secondary Outcome	Pilbery at al. [3]	Fiore et al. [12]	Jensen et al. [13]	Lin et al. [14]	DuCanto et al. [15]	Guillote et al. [16]	Frantz et al. [17]	Conclusion
Number of Attempts ↓	✖	✔ (1 attempt)	✔ (0 failures post)	✔ (post-training)	✖	✔ (1 attempt)	✖	The number of attempts decreased post-training.
Skill Retention at Follow-up ↑	✖	✖	✔ (3-month retention)	✖	✖	✖	✖	Skill retention evaluated by only one study but reported maintained performance at 3 months.
Confidence Improvement ↑	✖	✖	✔ (qualitative)	✔ *p* < 0.05	✔ *p* < 0.05	✖	✖	Confidence improved significantly.

(✔ = Outcome improved significantly (based on reported data). ✖ = Outcome not improved, not assessed, or not statistically significant. “↑/↓” indicates the direction of change. ↑ = Desired outcome increased (e.g., success rate, confidence). ↓ = Desired outcome decreased (e.g., intubation time, contamination). Where available, *p*-values or descriptive notes are included for clarity on statistical significance).

**Table 3:** Summary of findings and certainty of evidence table.

Study Design (Majority)	ROB	Inconsistency	Indirectness	Imprecision	Publication Bias	Effect (Direction)	Certainty of Evidence
First-pass intubation success							
Simulation (observational)	Serious	Not serious	Serious	Serious	Not serious	Improves success rate	⨁◯◯◯ Very Low
Time to Intubation							
Simulation (observational)	Serious	Not serious	Serious	Serious	Not serious	Reduced intubation time	⨁◯◯◯ Very low
Visualization Quality							
Simulation (observational)	Serious	Not serious	Serious	Serious	Not serious	Improved visualization	⨁◯◯◯ Very low
Aspirate Volume							
Simulation (observational)	Serious	Not serious	Serious	Serious	Not serious	Reduced contamination (qualitative)	⨁◯◯◯ Very low
Adverse Events/ Complications							
Case Reports	Serious	Not serious	Serious	Very serious	Not serious	No complications reported	⨁◯◯◯ Very low
Number of Intubation Attempts							
Simulation (observational)	Serious	Not serious	Serious	Serious	Not serious	Fewer attempts post-training	⨁◯◯◯ Very low
Skill Retention							
Simulation (observational, 1 study)	Serious	Not serious	Serious	Very serious	Not serious	Maintained skills at 3 months	⨁◯◯◯ Very low
Provider Confidence							
Simulation (observational)	Serious	Not serious	Serious	Serious	Not serious	Confidence improved	⨁◯◯◯ Very low

Certainty symbols: ⨁◯◯◯ = very low certainty. Domain Judgments: Not serious—no important concerns in this domain; no downgrade applied. Serious—important concerns that lower confidence; downgrade by 1 level. Very serious—major concerns that substantially lower confidence; downgrade by 2 levels.

## Data Availability

Not applicable. No new data were created or analyzed in this study. Data sharing is not applicable to this article.

## Supplementary Materials

The following supporting information can be downloaded at: https://www.mdpi.com/article/10.3390/jcm14207430/s1, Table S1: Detailed summary of the Study Characteristics.

## Abbreviations

SALAD	Suction-Assisted Laryngoscopy and Airway Decontamination
ED	Emergency department
PRISMA	Preferred Reporting Items for Systematic Reviews and Meta-analyses
CAMT	Cadaveric airway management training
CICO	Can’t Intubate, Can’t Oxygenate
SATIATED	Soiled Airway Tracheal Intubation And The Effectiveness of Decontamination
RSC	Rigid Suction Catheter
RCT	Randomized Controlled Trials
ROB2	Risk of Bias 2
CARE checklist	Consequences, Alternatives, Reality, External factors
HEMS	Helicopter Emergency Medical Service
IEI	Intentional Esophageal Intubation
