# Supplementary material for: Suction-Assisted Laryngoscopy and Airway Decontamination (SALAD) for Emergency Airway Management: A Systematic Review of Evidence and Implementation
Source: J Clin Med. 2025 Oct 21;14(20):7430. doi: 10.3390/jcm14207430 (PMC12564951; doi:10.3390/jcm14207430)
Supplement: Supplementary file 1 [file jcm-14-07430-s001.zip › jcm-3868056-supplementary.pdf]

Table S1: Detailed summary of the Study Characteristics

Study Identification								Study Characteristics				Population			
**ID**	**DOI**	**Author**	**Publication year**	**Title**	**Country / Institution**	**Funding source**	**Conflict of interest (decalred/nondeclared)**	**Study design**	**Setting**	**Simulation or clinical**	**Blinding and randomization (yes/o/notstated)**	**Participant Type**	**Number of Participants**	**Participant Completion / Adherence**	**Target Patient Type**
1	10.1016/j.amj. 2019.10.005	Matthew Jensen et al.	2020	Impact of Suction-Assisted Laryngoscopy and Airway Decontamination Technique on Intubation Quality Metrics in a Helicopter Emergency Medical Service: An Educational Intervention	USA / Virginia Commonwealth University	Biostatistics Consulting Laboratory, NIH (UL1TR002649)	Not Declared	Prospective before-and-after educational intervention	Helicopter EMS / Critical care transport training	Simulation	Not blinded, no randomization	Critical care transport providers (nurses + paramedics)	25 recruited; 20 completed full study	80% (20/25 completed all sessions)	Simulated massively soiled airway (not actual patients)
2	10.5811/westjem. 2016.9.30891	James DuCanto, Karen Serrano, Ryan Thompson	2016	Novel Airway Training Tool that Simulates Vomiting: Suction-Assisted Laryngoscopy Assisted Decontamination (SALAD) System	USA<br><br>Aurora St. Luke's Medical Center, University of North Carolina, University of Wisconsin	Not Declared	Not Declared	Educational pilot study with pre- and post-survey	Academic medical centers	Simulation	NA	Mixed healthcare providers (EM physicians, anesthesiologists, RNs, CRNAs, paramedics, RTs, students)	40	100% (all completed surveys)	Simulated vomiting/hemorrhaging airway scenario
3	10.1080/10903127. 2024.2360688	Guillote CP, Root CW, Braude DA, Decker CA, Romero AP, Perez NE, DuCanto JC	2024	Prehospital SALAD Airway Technique in an Adolescent with Penetrating Trauma Case Report	USA / Harris County EMS, Baylor College of Medicine	Not stated	Declared. One author (J.C. DuCanto) is the inventor of the DuCanto suction catheter (sold by SSCOR)	Case report	Prehospital	Clinical	NA	EMS patient Adolescent	1	1/1 completed	Adolescent with penetrating maxillofacial trauma (gunshot wound to mouth) and massive oropharyngeal bleeding.
4	10.29045/14784726. 2019.06.4.1.14	Richard Pilbery, M Dawn Teare	2019	Soiled airway tracheal intubation and the effectiveness of decontamination by paramedics (SATIATED): a randomised controlled manikin study	UK	Industry	None	RCT	Simulation lab	Simulation	Randomised only	Paramedics	154	154/154 100% adherence	Adults
5	10.1097/MD. 0000000000017898	Lin L-W, Huang C-C, et al.	2019	The S.A.L.A.D. Technique Toward Successful Intubation During Massive Vomiting Simulation – A Pilot Before–After Study	Taiwan / Shin Kong Wu Ho-Su Memorial Hospital	Not declared	Not declared	Pilot prospective before-after simulation study	Simulation (manikin-based training sessions using a SALAD vomiting simulator)	Simulation	Not blinded, no randomization	EMT paramedics	41	100% adherence	Simulated adult patient with massive vomiting (soiled airway scenario)
6	10.5811/westjem. 2019.6.42222	Michael P Fiore, Steven L Marmer, Michael T Steuerwald, Ryan J Thompson, Richard E Galgon	2019	Three Airway Management Techniques for Airway Decontamination in Massive Emesis: A Manikin Study	USA	Industry	Not declared	Prospective, randomized, crossover manikin study	Simulation lab	Simulation	Randomized only	Physicians and residents	35	35/35, 100% adherence	Adults
7	10.1002/emp2.12344	Eric Frantz, MD<br>Nima Sarani, MD<br>Andrew Pirotte, MD<br>Bradley S. Jackson, MD	2021	Woman in respiratory distress	USA	NA	Not declared	Case report	ED	Clinical	Not blinded, no randomization	Patient	1	NA	NA

Intervention: SALAD Technique						Comparator				Outcomes Measured - Primary							
**Was SALAD Used? (Yes/No)**	**SALAD Implementation Type**	**Device/Equipment Used**	**SALAD Training Included? (Yes/No)**	**Training Duration / Content**	**Instructor Type**	**Comparator Design Type**	**Comparator Description**	**Comparator Provider Experience**	**Baseline Conditions (If applicable)**	**First-pass time to successful intubation**	**Timing of first-pass time measurement**	**Quality of visualization**	**Timing of quality measurement**	**Aspirate Volume / Airway Contamination**	**Timing of aspirate Volume measurement**	**Adverse events reported**	**Timing of adverse events measurement**
Yes	Hands-on training with demo	C-MAC video laryngoscope, SSCOR DuCanto catheter	Yes	20-minute demo and Q&A by board-certified EM physician	EMS-trained, board-certified emergency physician	Within-subject (pre/post)	Intubation of vomiting mannequin without SALAD technique	Mixed (nurses + paramedics, likely experienced)	Vomiting high-fidelity mannequin without SALAD	Overall median times:<br>Pre-instruction: 60.5 sec (IQR 44.0–84.0)<br>Post-instruction: 43.0 sec (IQR 38.0–57.5)<br>3-month follow-up: 29.5 sec (IQR 24.5–39.0)<br>Bottom 50th percentile:<br>Pre: 84.0 sec (IQR 68.0–96.0)<br>Post: 41.5 sec (IQR 36.0–65.0)<br>3-month: 29.5 sec (IQR 25.0–39.0)<br>Top 50th percentile:<br>Pre: 44.0 sec (IQR	Measured at:<br>Before SALAD instruction<br>Immediately after instruction<br>3-month follow-upc	No	N/A	No	N/A	No	N/A
Yes	Hands-on simulation session with vomiting mannequin	Modified Nasco airway head, fluid pump system, C-MAC video laryngoscope	Yes	Two simulated scenarios: static vomit and continuous emesis; suction and intubation training	Not specified	No explicit comparator group	Pre/post self-assessment (within-subject design)	Mixed (only 1 participant had prior emesis-based airway simulation experience)	None stated explicitly; baseline is pre-training confidence	No actual intubation timing recorded	N/A	C-MAC used to visualize glottic structures; quality not rated or scored	During simulation only	Simulated via continuous or static vomit (SAC)<br>No volume quantitatively measured	N/A (visual effect only)	No	N/A
Yes	Emergency prehospital application	VL (video laryngoscopy), SSCOR DuCanto catheter	NA	NA	NA	None	NA	NA	Massive bleeding and pharyngeal trauma	Not timed; successful on first attempt	NA	Maintained via continuous suction	During intubation	Several hundred ml of Emesis	Orogastric tube post intubation	No	During intubation
Yes	Simulation and hands on	Modified suction catheter and manikin	Yes	15 minutes, lecture and hands on simulation	Not stated	Parallel group	Traditional suctioning with a Yankauer suction catheter	Experienced providers	standard practice without SALAD training	29.2 seconds (mean)	Post training	Score (Cormack-Lehane (C-L) grading)	Post training	11.0 mL	Post training	No	None
Yes	Hands on and simulation	Modified Nasco airway mannequin, video laryngoscope, drill pump, Yankauer suction	Yes	1-hour session with lecture, demo, and hands-on	Emergency physician	Pre/post	Three scenarios: (1) Control = clean airway; (2) Pre-training = vomiting airway, before SALAD training; (3) Post-training = vomiting airway, after SALAD training	Experienced	Clean airway (control) and pre-training in soiled scenario	Control: 12s; Pre: 37.1 s; Post: 26.9s (median)	Pre/post; each attempt recorded	NA	NA	Simulated emesis at 237 mL/s	NA	No	NA
Yes	Hands on and simulation	Standard suction cathter and manikin	Yes	5 minutes, hands on simulation	Not stated	Crossover	Traditional suction, SALAD and intentional esophageal intubation with suctioning	Mixed	Standard practice without SALAD, representing usual care with traditional suction techniques before SALAD training	35 seconds (median) for SALAD	Post training	Subjective rating (poor to good)	Post training	None reported, visually estimated. SALAD was shown to better clear the airway	Post training	No	None
Yes	Clinical application	Modified suction catheter	NA	NA	NA	None	NA	NA	NA	NA	NA	Subjective	Post SALAD procedure	Described as "cleared enough to allow for successful visualization and intubation."	Post SALAD procedure	No	NA

		Outcomes Measured - Secondary						Results			Qualitative feedback/ User experience			Quality and Bias			Notes & Reviewer Input		
**Success Rate in Soiled Airway**	**Timing of success rate measurement**	**Number of Attempts**	**Timing of attempts measurement**	**Skill Retention at Follow-up**	**Timing of skill retention measurement**	**Confidence Improvement (Pre/Post Training)**	**Timing of confidence improvement measurement**	**Quantitative Results**	**Statistical Significance**	**Subgroup Results**	**Provider Feedback or Confidence Scores**	**Usefulness & Realism Ratings**	**Reported Challenges**	**Quality assesment tool used**	**Risk of bias score/summary**	**Study limitations**	**Reviewer Comments**	**Relevance to Review Objective**	**Other Techniques Compared**
Pre-training: 90% (18/20)<br>Post-training & 3-month: 100% (20/20)	Same 3 stages: pre, post, and 3-month follow-up	Pre: 2 participants needed >1 attempt<br>Post and follow-up: 100% success on first attempt	All three timepoints	Yes	At 3-month follow-up	Indirectly (not formally scored but inferred through improved performance and retention)	N/A	Median time to intubation:<br>Overall: ↓ from 60.5 sec to 29.5 sec<br>Bottom 50% subgroup: ↓ by ~63 sec<br>First-pass success improved to 100%<br>Statistically significant improvements	Yes<br>e.g., overall pre vs 3-month: P < .001<br>Bottom 50% pre vs 3-month: P < .001	Bottom 50% benefited most (↓62.9 sec in intubation time)<br>Top 50% had smaller but significant improvement by 3 months	Not directly collected as scores<br>Reported improved ease and conceptual understanding	Not numerically rated, but:<br>High-fidelity mannequin with realistic emesis simulation described<br>Realistic SALAD training scenario with EMS physician-led instruction	Simulation not reflecting real-world stress<br>Potential priming effect<br>Small sample size<br>Some dropout at follow-up (5 lost to 3-month data)	Robins 1	Serious risk	Small N<br>Sim environment doesn't reflect real-life stress<br>No clinical outcome data<br>Possible Hawthorne effect or simulation priming	Simulation-only data limits generalizability.<br>High relevance for prehospital training environments.<br>Suggests SALAD is most beneficial for those initially struggling with soiled airways.<br>Retention at 3 months is encouraging for long-term skill maintenance.	Highly relevant for assessing SALAD's impact on airway management performance.<br>Directly evaluates intubation metrics, first-pass success, and skill retention using SALAD.	No comparison to other airway adjuncts or techniques
Not measured in terms of intubation success	N/A	Not tracked	N/A	Not assessed; no follow-up testing performed	N/A	Yes:<br>Managing vomiting/bleeding airway:<br>Pre: 3.10 ± 0.49<br>Post: 4.13 ± 0.22<br>Suction skills:<br>Pre: 3.30 ± 0.43<br>Post: 4.03 ± 0.26	Immediately before and after the session	Confidence improved<br>Usefulness of session: 4.68 ± 0.15<br>Simulator realism: 4.65 ± 0.17	Yes (statistically significant improvement in confidence reported)	Not reported	Confidence Scores improved significantly<br>Majority planned to apply SALAD technique in clinical practice (mean score: 4.53 ± 0.19)	Usefulness: 4.68 ± 0.15<br>Realism: 4.65 ± 0.17	No clinical outcomes measured<br>No long-term retention assessed<br>Multidisciplinary group limits role-specific conclusions<br>Small sample	Robins 1	Serious risk	Small sample<br>No clinical validation<br>Confidence may not equal competence<br>Skill decay possible (no follow-up)<br>Results not generalizable across provider types	Promising as a low-cost, high-realism simulation<br>Excellent for introducing SALAD technique<br>Lacks hard clinical endpoints<br>Needs future validation in real-patient settings	Highly relevant to simulation-based SALAD training outcomes<br>Supports use in multidisciplinary airway education	None directly compared to SALAD in this study
100%	Immediate during procedure	1	During procedure	NA	NA	NA	NA	Case based	NA	NA	NA	NA	Bleeding control, airway clearance	CARE checklist used	Moderate risk (score 11); scores >11 indicating low risk, 7–11 moderate risk, and ≤6 high risk of bias.	Single case, anecdotal	Demonstrates real-world success of SALAD technique. This case reports the use SALAD technique in a prehospital setting by EMS personnel to manage patient with severe facial trauma and profuse airway bleeding. Using video laryngoscopy and ongoing suction, the airway was secured on the first attempt without any complications. Although this example highlights the potential of SALAD in real-world, high-stakes scenarios, the findings are limited by its	Highly relevant as a real-life clinical example	Standard VL, direct laryngoscopy, surgical airway (backup only)
0.974	Post training	first pass attempt measured, total number unclear	Post training	Not assessed	Not assessed	Not assessed	Not assessed	Number of paramedics per group (control/salad)= 77. First pass intubation time= 29.2 seconds (SALAD), 37 seconds (control). Residual aspirate volume= 11mL (SALAD), 50.3mL (control). Cormack-Lehane grade 1 or 2= higher (SALAD), lower (control).	SALAD vs control: first pass intubation time p<0.001, first pass success rate p<0.001, residual aspirate volume p<0.001, Cormack Lehane grade p<0.001	No subgroups results	None reported	None reported	None reported	Robins 2	Some concerns	1. Simulation-based, real life is more complex. 2. No blinding. 3. Short training. 4. Paramedics from single centre	The SATIATED study randomized 154 paramedics to SALAD training or standard airway management in a simulated soiled airway. SALAD training significantly improved first-pass intubation success (97.4% vs. 82.5%), reduced intubation time (29.2s vs. 37.0 s), and decreased airway contamination	High	Standard suction with Yankauer catheter (control group)
Pre: 82.9%, Post: 92.7%	Immediately pre vs post training	Each participant, 3 attempts per scenario	Each simulation session pre/post training	Not assessed	NA	endotracheal intubation in clean airway 82.9% →100%; soiled-airway intubation 22.0%→97.6%; use of suction instruments 46.3%→92.7% (pre- vs post-training)	Pre/ post training	Intubation time: Control 12.0s; Pre-training 37.1s; Post-training 26.9s Success counts: 3/3-success: Control 92.7%, Pre 36.6%, Post 80.5%; Pass rates: Control 100%, Pre 82.9%, Post 92.7%	Yes. Intubation time was significantly shorter post-training vs pre-training (P=0.031) ; success rates improved (Pre vs Post, P=0.001) . Confidence improvements were also statistically significant (p<0.05 for key items).	Trainees that did not pass pre training all succeeded post-training. Their post-training intubation times (median 31.5 s) were similar to those who had succeeded pre-training (26.3 s)	Very positive; 75.6% gave highest rating	Yes	Vomitus contamination was the most common cause of failed intubation in practice (46.3% cited)	Robins 1	Critical risk	Small sample size (n=41 volunteers), all EMT-Ps from 2 divisions; non-randomized design; possible learning effect from repeated attempts; simulation (vomit manikin) may not fully replicate clinical conditions	This pre/post training simulation study evaluated SALAD training in 41 EMT-paramedics using a vomiting manikin. Post-training, intubation time significantly decreased (37.1s to 26.9s), and pass rates improved (82.9% to 92.7%). Confidence in	High	Compared standard laryngoscopy (control) vs. SALAD-assisted technique.
0.97	Post training	One attempt	Post training	Not assessed	Not assessed	Not assessed	Not assessed	Time to first pass successful intubation= 35seconds (SALAD), 45 seconds (traditional suction), 50 seconds (intentional esophageal suction). First pass success rate= 97% (SALAD), 91% (traditional suction), 88% (intentional esophageal suction). Airway contamination= less residual vomit with SALAD. Visualisation quality (Likert)= SALAD scored higher	SALAD had faster intubation times p<0.05. SALAD had higher first pass success rates p<0.05. Better airway visualisation with SALAD was also statistically significant	No subgroups results	None reported besides verbal comments during debriefing praising SALAD technique	Usefulness and realism were assessed through subjective Likert-scale ratings, SALAD scored significantly higher on both, ratings weren't specified	Some found SALAD initially tricky. Managing simultaneous suction and laryngoscopy required practice.	Robins 2	Low	1. Simulation based. 2. Small sample size. 3. Short training. 4. No blinding. 5. Subjective outcome measurement	Good randomised simulation study showing SALAD improves speed and success in soiled airways. Limited by small sample, simulation setting, brief training, and no skill retention data. Real-world studies needed.	High	Traditional suction with Yankauer, SALAD, intentional esophageal intubation with suctioning
NA	NA	NA	NA	NA	NA	NA	NA	NA	NA	NA	NA	NA	None reported	CARE checklist used	Moderate	1. Single report. 2. Lack of quantitative data. 3. No control for comparison. 4. Limited details (follow up). 5. Possible publication bias	highlights the practical utility of the SALAD technique in managing a heavily contaminated airway during emergency intubation. It provides clear clinical insights and reinforces the importance of airway suctioning skills. However, as a single case	Moderate	None
